# AI for Causality Assessment in Pharmacovigilance: Protocol for a Scoping Review

**DOI:** 10.2196/101691

**Published:** 2026-07-16

**Authors:** Miki Ohta, Miki Ota, Mikihiko Ohta

**Affiliations:** 1 Clinical Research Promotion Center The University of Tokyo Hospital Tokyo Japan; 2 Office of Informatic and Management for Safety Pharmaceuticals and Medical Devices Agency Tokyo Japan; 3 Fuji Partnership Law Firm Tokyo Japan

**Keywords:** artificial intelligence, AI, pharmacovigilance, causality assessment, machine learning, scoping review

## Abstract

**Background:**

Pharmacovigilance aims to protect patient safety by identifying and managing adverse events associated with pharmaceuticals. Determining the causality of these adverse events is central at both the individual case and population levels; however, it is increasingly challenging as the volume and complexity of safety data grow. Although AI and related technologies have been proposed to support causality assessment, limited research has examined how these methods are used, their information and quality requirements, or how associated risks are addressed.

**Objective:**

This scoping review aims to determine the available evidence on AI-based methods for causality assessment in pharmacovigilance. The primary objective is to characterize how these methods are applied or proposed with a focus on their functional roles, reported data inputs and information needs, and associated risks. Secondary objectives include comparing applications at the individual case and population levels; describing the types of AI-based techniques and automation tools used in causality assessment workflows; and summarizing reported data quality considerations and governance mechanisms, including risk management approaches.

**Methods:**

Sources describing or proposing AI-based approaches, including data-driven models (machine learning, natural language processing, knowledge graphs, and causal inference) and knowledge- or rule-based systems implementing causal assessment logic, will be eligible. Searches will be conducted in PubMed, Web of Science Core Collection, ProQuest, EBSCOhost, and Ichushi Web and will be restricted to English- and Japanese-language sources. Two reviewers will independently screen records and full-text articles, with disagreements resolved by a third reviewer. Data will be charted on use cases, information inputs, data quality dimensions, model characteristics, governance mechanisms, and identified risks. Synthesis will follow a reflexive thematic analysis approach and be reported in accordance with PRISMA-ScR (Preferred Reporting Items for Systematic reviews and Meta-Analyses extension for Scoping Reviews) guidelines informed by applicable PRISMA-S (Preferred Reporting Items for Systematic reviews and Meta-Analyses literature search extension) elements.

**Results:**

This protocol was registered in the Open Science Framework platform on December 23, 2025. The registration was subsequently updated on May 19, 2026, to reflect an extension to the data collection period. A preliminary database search was conducted in December 2025, retrieving a total of 760 records, of which the preliminary title and abstract screening identified 196 (25.8%) articles for full-text review. Database searches are scheduled for July 2026. Data charting is scheduled for August 2026, and synthesis is scheduled for September 2026. Findings are expected to be submitted for publication by the end of December 2026.

**Conclusions:**

This review is expected to provide a structured map of AI-based applications for causality assessment in pharmacovigilance, clarify reported information inputs and data quality dimensions, and synthesize risk management and governance approaches. The findings are expected to inform methodological development, practical implementation, and the governance of AI-supported causality assessment.

**Trial Registration:**

Open Science Framework 10.17605/OSF.IO/QVF5C; https://osf.io/qvf5c/overview

**International Registered Report Identifier (IRRID):**

DERR1-10.2196/101691

## Introduction

Pharmacovigilance aims to protect patient safety by detecting, assessing, understanding, and preventing adverse events associated with medicinal products [1]. A central activity in pharmacovigilance is the assessment of causal relationships between a drug and an adverse event at both the individual-case and population levels [2]. At the individual-case level, causality assessment is conducted in clinical settings, by regulatory authorities, and by the pharmacovigilance departments of pharmaceutical companies, most often through clinical judgment alone or in combination with standardized tools (eg, the World Health Organization Uppsala Monitoring Centre scale [3] and the Naranjo algorithm [4]). At the population level, statistical and epidemiological methods such as disproportionality analyses and observational studies support signal detection and benefit-risk assessment [5].

The rapid expansion in both the volume and complexity of safety data, driven by electronic reporting, large numbers of spontaneous reports, and diverse real-world data sources, has made timely and consistent causality assessment increasingly difficult to achieve through manual review alone. Accordingly, interest has grown in the use of advanced IT, particularly AI, sometimes in combination with automation tools such as robotic process automation to support and potentially transform causality assessment in pharmacovigilance.

Recent developments in AI, including machine learning, natural language processing, knowledge graphs, and causal inference methods, have been explored for various pharmacovigilance tasks such as automated coding [6], duplicate detection [7], case triage, signal detection and prioritization [8], and literature screening [9]. Concurrently, robotic process automation has been introduced to automate repetitive tasks such as case intake, data transfer, and report generation [10]. Although many of these applications primarily aim to improve efficiency and consistency, AI-based models are increasingly being developed to contribute directly to causality assessment—for example, by predicting causality categories for individual cases [11] or identifying patterns indicative of causal associations in large databases [12].

The application of AI-based methods to causality assessment raises several methodological, operational, and governance issues [11,13,14]. These systems must integrate structured and unstructured data at the individual case level and heterogeneous real-world data at the population level, often supplemented by external knowledge databases [11,14]. In addition, pharmacovigilance databases are affected by missing data, underreporting, misclassification, and reporting bias, all of which may influence AI performance [15-17]. Consequently, data quality metrics and performance indicators beyond standard measures of accuracy must be considered to ensure that outputs align with clinically and regulatorily meaningful outcomes. Explainability and human-in-the-loop designs also remain essential as ultimate decisions regarding causality and safety actions continue to rest with experts [18-20]. Furthermore, AI-based systems and associated automation introduce risks such as algorithmic bias, model drift, overfitting, automation bias, and privacy and security concerns, which may manifest differently at the individual case and population levels and require tailored governance approaches [13,18,21-23].

In response to these emerging applications and challenges, international and national organizations, including the Council for International Organizations of Medical Sciences, the European Medicines Agency, and the US Food and Drug Administration, have begun to articulate principles for the use of AI across the medicinal product life cycle [20,23,24]. These initiatives emphasize data quality, model validation, explainability, human oversight, and governance throughout the AI life cycle.

Despite these advancements, several knowledge gaps remain. First, existing guidance documents are largely high level and cross-cutting and rarely specify, in a concrete or systematic manner, the types of information required, the data quality thresholds that should be met, or the risk management approaches needed in this context. Second, although the literature on AI in pharmacovigilance is expanding, most reviews provide broad overviews of applications and do not focus specifically on causality assessment, nor do they systematically distinguish between individual case– and population-level applications. Third, there is limited synthesis regarding which AI techniques and related automation tools are used at specific stages of causality assessment, the information and data quality assumptions on which they rely, and how associated risks are identified and addressed. Finally, the interplay and potential complementarity between AI-supported processes at the individual case and population levels remain insufficiently described.

Given this combination of emerging applications and persistent knowledge gaps, a scoping review is an appropriate approach for mapping the existing evidence, clarifying key concepts, and identifying areas requiring further research. Rather than quantifying the effect of a specific intervention, this review will provide an overview of how AI-based methods and, where applicable, AI-enabled automation are currently used, proposed, or discussed for causality assessment in pharmacovigilance. It will also summarize reported information inputs, data quality considerations, and associated risks. A preliminary search of MEDLINE (via PubMed), the Cochrane Database of Systematic Reviews, and *JBI Evidence Synthesis* identified no current or ongoing systematic or scoping reviews on this topic.

The main objectives of this study are described in Textbox 1.

**Objectives of this scoping review.**

**Primary objective**

- Characterize how AI-based methods are applied or proposed for causality assessment between medicinal products and adverse events in pharmacovigilance, with a focus on their functional roles, reported data inputs, information needs, and associated risks

**Secondary objectives**

- Compare how these AI-based approaches differ between individual case–level and population-level applications, including potential complementarities between the 2 levels
- Describe the types of AI-based techniques and supporting automation tools used in causality assessment workflows
- Summarize reported data quality considerations and governance mechanisms (eg, human oversight, explainability, monitoring, and model maintenance) that support the safe use of AI in this context

## Methods

### Overview

A scoping review methodology was selected to map concepts, approaches, and evidence within a heterogeneous and rapidly evolving field rather than to estimate effect sizes or conduct a meta-analysis. This protocol follows guidance from the *JBI* *Manual for Evidence Synthesis* [25] and will be reported, as applicable, in accordance with PRISMA-ScR (Preferred Reporting Items for Systematic Reviews and Meta-Analyses extension for Scoping Reviews) [26] and PRISMA-S (Preferred Reporting Items for Systematic Reviews and Meta-Analyses extension for reporting literature searches in systematic reviews) [27]. The PRISMA-ScR and PRISMA-S checklists are provided in Multimedia Appendices 1 and 2, respectively. The protocol was prospectively registered in the Open Science Framework [28].

### Operational Definitions

#### Causality Assessment

In this review, causality assessment is defined as the plausibility evaluation of a causal relationship between a drug and an observed adverse event through the structured, qualitative synthesis of all available evidence streams [2]. This definition encompasses both individual case–level assessment, in which evidence from a specific report or patient is evaluated, and population-level assessment, in which evidence from aggregated safety data, real-world data, or epidemiological analyses is used to evaluate potential causal associations or safety signals.

Population-level causality assessment refers specifically to the evaluation of causal plausibility at the population level. This includes the explicit causal interpretation of detected safety signals, such as signal evaluation or validation, confounding assessment, causal modeling, or structured benefit-risk reasoning. In contrast, routine signal detection based solely on frequency-based disproportionality measures without causal interpretation is not considered population-level causality assessment and is excluded.

#### AI-Based Methods

AI-based methods are operationally defined as computational approaches that use data-driven learning, natural language processing, knowledge representation, causal modeling, or explicit knowledge- or rule-based reasoning to support or perform causality assessment. These include but are not limited to the following: (1) data-driven approaches such as machine learning models, deep learning models, natural language processing models, knowledge graph–based approaches, and causal inference models; (2) knowledge- or rule-based systems, including expert systems, that explicitly implement causal assessment logic, structured causality criteria, or rules for evaluating drug-event relationships; and (3) hybrid systems that combine statistical, machine learning, natural language processing, and knowledge- or rule-based components.

#### AI-Based Causality Assessment

AI applications relevant to causality assessment can be classified into 4 categories, as shown in Textbox 2. The applications covered in this review fall into categories 1 to 3, which are distinguished from upstream support functions and pure business automation (category 4).

**AI application categories.**

**Category 1: AI used for extracting or structuring information relevant to causality assessment**

This category is limited to tools that extract, structure, or synthesize evidence elements explicitly relevant to causal review, such as temporal relationships between exposure and event, de- or rechallenge information, dose-response information, alternative explanations, concomitant medications, comorbidities, relevant laboratory or clinical findings, and prior evidence. General information extraction, coding, or data processing will not be considered sufficient for inclusion unless the extracted information is explicitly used or intended to be used to support causal judgment or structured causality assessment.

**Category 2: AI used to assign or predict individual case–level causality or relatedness categories**

This category includes systems that support or automate the application of structured causality criteria or assign relatedness categories for observed adverse events at the individual case level. AI used exclusively for adverse event forecasting (ie, estimating the likelihood of adverse events occurring in patients who have not yet experienced them) is outside the scope of this review.

**Category 3: AI used for population-level causal inference or signal evaluation**

This category includes methods that explicitly address causal interpretation, confounding, counterfactual reasoning, causal modeling, signal evaluation, or totality-of-evidence assessment in relation to a potential drug-event association. The use of frequency-based disproportionality measures alone or the term “signal detection” alone will not be sufficient for inclusion unless the method is explicitly linked to causal interpretation, confounding assessment, signal evaluation, causal inference, or totality-of-evidence assessment.

**Category 4: AI or automation used solely for operational efficiency**

This category includes tools used for case intake, routing, duplicate detection, Medical Dictionary for Regulatory Activities coding, data transfer, report generation, workflow management, or administrative prioritization when they do not incorporate any causal reasoning component or explicit linkage to causal evidence synthesis.

Causality assessment algorithms such as the World Health Organization Uppsala Monitoring Centre system or the Naranjo algorithm will not be considered AI-based methods when they are used only as manually applied criteria or as simple automated score calculators. They will be considered within scope only when AI- or knowledge-based methods automate the interpretation or application of the criteria, for example, by extracting relevant information from case narratives or other evidence sources and applying the criteria to support a causality judgment.

Simple statistical models such as logistic regression will be included only when explicitly implemented as a causal model or as part of a rule-based system for causality assessment. Models used solely for outcome prediction, prioritization, or classification without explicit causal framing will be excluded. Similarly, conventional descriptive statistics, disproportionality analyses used only as frequency-based screening without causal interpretation, and noncausal workflow automation will not be considered AI-based causality assessment by themselves.

For methods that fall near the eligibility boundary, inclusion will be determined by whether they are explicitly used to support a causal judgment regarding the relationship between a medicinal product and an adverse event.

### Eligibility Criteria

The review will address the population, concept, and context framework recommended by the JBI for scoping reviews [25].

The central concept of interest is the use of AI-based methods to support or perform causality assessment for relationships between medicinal products for human use, including vaccines and biologics, and adverse events at either the individual case or population level. Medical devices, as well as device components of drug-device combination products, are excluded; studies concerning the medicinal component of such products remain eligible. At the population level, causality assessment is distinguished from routine signal detection: it encompasses approaches that address confounding or bias, apply causal inference methods, or provide explicit causal interpretation of a signal.

Sources that describe or evaluate systems, methods, or workflows in which AI-based methods directly contribute to causality assessment or its interpretation in pharmacovigilance; propose frameworks, requirements, or governance models specifically for AI-based causality assessment in pharmacovigilance; or both will be included.

The context of interest is pharmacovigilance and medicinal product safety for human use, encompassing marketing authorization holders and pharmaceutical companies, regulatory agencies and public health authorities, health care settings (eg, hospitals and clinics), and academic or collaborative research networks conducting pharmacovigilance or pharmacoepidemiological studies. No restrictions will be placed on country, health system, or regulatory framework.

Nonempirical publications, including opinion pieces, editorials, and narrative commentaries that do not include substantive descriptions of AI-based methods or their application to causality assessment will be excluded. The complete eligibility criteria are summarized in Table 1.

**Table 1:** Eligibility criteria (population, concept, and context framework).

Category	Inclusion criteria	Exclusion criteria
Population and data sources	Human pharmacovigilance or drug safety–related data, including individual case safety reports and spontaneous reports; electronic health records and hospital information systems; disease or drug registries; pharmacoepidemiological datasets; clinical trial safety datasets; published literature for safety assessment; and social media, patient forums, and direct patient reports used for pharmacovigilance purposes	Studies focusing exclusively on veterinary pharmacovigilance and sources not involving medicinal product safety, adverse events, pharmacovigilance, pharmacoepidemiology, or drug safety–related evidence
Concept	AI-based methods directly contributing to causality assessment, including (1) data-driven approaches (machine learning, deep learning, natural language processing, knowledge graphs, and causal inference models) and (2) knowledge- or rule-based systems explicitly implementing causal assessment logic. Both individual case– and population-level causality assessment are eligible. Eligible applications include AI used to extract or structure causality-relevant evidence elements, assign or predict case-level causality or relatedness categories, or support population-level causal inference or signal evaluation with explicit causal interpretation. Frameworks, requirements, or governance models for AI-based causality assessment in pharmacovigilance are also eligible.	AI applied only to pharmacovigilance tasks unrelated to causality assessment with no explicit causal link, including general information extraction, MedDRA^a^ coding, duplicate detection, case routing, or workflow automation without extraction or structuring of causality-relevant evidence elements; automation-only tools without an AI component; simple automated scoring of causality algorithms without AI-enabled evidence interpretation or application; statistical or prediction models without explicit causal framing; frequency-based disproportionality analyses or “signal detection” with no explicit causal interpretation, confounding assessment, signal evaluation, causal inference, or totality-of-evidence assessment; and AI used solely for purposes unrelated to drug safety or pharmacovigilance
Context	Human pharmacovigilance and medicinal product safety settings, including marketing authorization holders; pharmaceutical companies; regulatory agencies; public health authorities; health care settings; and academic or collaborative research networks conducting pharmacovigilance, pharmacoepidemiological, or drug safety research, with no restrictions based on country, health system, or regulatory framework	Veterinary pharmacovigilance; AI applications used solely outside pharmacovigilance or medicinal product safety, such as marketing, adherence prediction, general clinical decision support unrelated to safety, or nonsafety administrative processes; and medical devices (including device-only vigilance) and combination products with respect to their device component
Types of sources	Quantitative, qualitative, and mixed methods empirical studies; methodological or technical papers; evaluation, feasibility, and pilot implementation studies; case studies; regulatory or industry reports with sufficient methodological details; and theses and conference proceedings with sufficient methodological details	Opinion pieces, editorials, or narrative commentaries without substantive descriptions of AI-based methods, requirements, or governance
Language and time frame	English or Japanese; no restriction based on publication year	All languages other than English or Japanese

^a^MedDRA: Medical Dictionary for Regulatory Activities.

### Information Sources

Searches will be conducted in the following bibliographic databases: (1) PubMed (MEDLINE), (2) Web of Science Core Collection (Science Citation Index Expanded, Social Sciences Citation Index, Arts and Humanities Citation Index, Emerging Sources Citation Index, Book Citation Index, Conference Proceedings Citation Index, Current Chemical Reactions, and Index Chemicus), (3) ProQuest, (4) EBSCOhost platform (Academic Search Complete, Bibliography of Asian Studies, Business Source Complete, CINAHL Plus with Full Text, Communication Abstracts, EconLit, Education Resources Information Center, GreenFILE, Historical Abstracts, Index to Legal Periodicals & Books, Information Science & Technology Abstracts, Linguistics Abstracts Online, MEDLINE, MLA Directory of Periodicals, MLA International Bibliography, Peace Research Abstracts, Philosopher’s Index, PsycArticles, PsycInfo, Race Relations Abstracts, Regional Business News, SocINDEX with Full Text, Teacher Reference Center, and American Antiquarian Society Historical Periodicals Collection: Series 1-5), and (5) Ichushi Web (Japanese medical literature database).

To identify additional sources not captured through database searches, the following supplementary strategies will be used. First, the reference lists of all included articles and relevant reviews or guidelines will be screened. Second, forward citation tracking may be conducted using Web of Science or Google Scholar where appropriate. Third, targeted searches of the websites of the following regulatory and organizational bodies will be conducted: the US Food and Drug Administration, the European Medicines Agency, the Council for International Organizations of Medical Sciences, the World Health Organization, the Uppsala Monitoring Centre, and the Pharmaceuticals and Medical Devices Agency. For each website, searches will use terms related to AI, pharmacovigilance, and causality assessment, focusing on guidance documents, reflection papers, and technical reports. The date of each search will be recorded.

The database searches will be limited to English and Japanese. Finally, no study design filter will be applied.

### Search Strategy

A 3-step search strategy as recommended by the JBI [25] will be used. The search strategy was developed a priori in consultation with an information specialist and will be reported in accordance with applicable elements of the PRISMA-S [27] adapted for the scoping review context in 3 steps.

First, the development of the final strategy (initial limited search): an initial limited search of PubMed and Web of Science was conducted using a small set of key terms related to AI, pharmacovigilance, and causality assessment. Titles, abstracts, and indexing terms (eg, MeSH terms and keywords) of relevant articles were examined to identify additional terms.

Second, the comprehensive database search: a full search strategy was developed for PubMed and adapted for each database, combining controlled vocabulary and free-text terms covering 3 main concept blocks (Table 2). All 3 blocks were combined using the Boolean operator AND; terms within each block were combined using OR.

Third, citation tracking and supplementary searches: the reference lists of all the included studies and key reviews or guidelines will be screened for additional relevant sources. Where appropriate, forward citation tracking may be conducted using Web of Science or Google Scholar. Targeted searches of relevant websites and repositories (as prespecified in the Information Sources section) will also be performed.

**Table 2:** Search concept blocks and representative terms (all 3 blocks will be combined using “AND”)^a^.

Block	Concept domain	MeSH terms or controlled vocabulary terms (PubMed)	Free-text keywords
1	Pharmacovigilance	“Pharmacovigilance” (MeSH), “Drug-Related Side Effects and Adverse Reactions” (MeSH), “Adverse Drug Reaction Reporting Systems” (MeSH), and “Product Surveillance, Postmarketing” (MeSH)	“Pharmacovigilance,” “drug safety,” “adverse drug reaction*,” “adverse drug event*,” “adverse reaction,*” “drug-related adverse event*,” “ADR,” “adverse event*,” “safety signal*,” “safety report*,” “spontaneous report*,” and “ICSR*”
2	Causality and causal assessment	“Causality” (MeSH), “Bayes Theorem” (MeSH), and “Models, Statistical” (MeSH)	“Causality assessment,” “causal inference,” “causal model*,” “causal relationship*,” “signal assessment,” “Naranjo,” and “WHO-UMC”
3	AI	“Artificial Intelligence” (MeSH), “Machine Learning” (MeSH), “Deep Learning” (MeSH), “Natural Language Processing” (MeSH), and “Expert Systems” (MeSH)	“Artificial intelligence,” “machine learning,” “deep learning,” “natural language processing,” “NLP,” “knowledge graph*,” “expert system*,” “prediction model*,” “generative AI,” “agentic AI,” “large language model*,” “LLM*,” “ChatGPT,” “Claude,” “Gemini,” “GPT-5*,” “GPT-4*,” and “GPT-3*”

^a^The PubMed database search strategy uses MeSH terms combined with free-text keywords in the title and abstract field. For the other information sources, the strategy was translated into database-appropriate field codes and, where available, controlled vocabulary terms while maintaining the same conceptual structure. Complete search strings for all 5 databases are provided in Multimedia Appendix 3.

### Handling of Japanese- and English-Language Records

As all members of the review team are fluent in both Japanese and English, records in both languages will be screened and charted in their original language. For Japanese-language records retrieved from Ichushi Web and other sources, title and abstract and full-text screening will be performed directly in Japanese by at least 2 reviewers. Data charting for Japanese sources will likewise be conducted based on the original Japanese texts, with key data items (eg, study design, data sources, AI methods, outcomes, and main conclusions) entered into the shared data charting form in English to facilitate integrated synthesis across languages. Where important concepts or terminology do not have a straightforward English equivalent, the original Japanese wording will be recorded in the data charting form alongside an English summary. Any uncertainties or disagreements regarding language interpretation will be resolved through discussion within the review team using the same multistep consensus process as that for other reviewer disagreements.

### Study and Source Selection

#### Overview

All search results will be exported to a reference manager (eg, EndNote; Clarivate Analytics), deduplicated, and then imported into a screening tool (eg, Rayyan; Rayyan Systems Inc) for study selection. When multiple publications describe the same AI system or implementation, they will be grouped as a single system unit during data charting. The most recent and most methodologically comprehensive publication will serve as the primary reference, with others consulted as supplementary sources. Descriptive statistics will report both the number of distinct systems identified and the total number of publications included.

Study selection will proceed in 2 stages as follows. First, for title and abstract screening, 2 reviewers will independently screen titles and abstracts against the eligibility criteria. Records clearly not meeting the inclusion criteria will be excluded. Records retained by either reviewer will proceed to the full-text screening stage. Second, for full-text screening, the full texts of potentially relevant records will be retrieved. Two reviewers will independently assess each full text against the inclusion and exclusion criteria. Reasons for exclusion at this stage will be recorded (eg, not a pharmacovigilance context, AI not used for causality assessment, or insufficient methodological details).

#### Calibration and Agreement

Before full screening, the reviewers will pilot the eligibility criteria on a sample of approximately 20 to 30 records to ensure shared understanding and refine operational definitions if needed.

#### Disagreement Resolution

At both the title and abstract and full-text stages, disagreements between the 2 reviewers will first be flagged within the screening tool. A prespecified third reviewer will then independently assess the record. If consensus is not reached after the third reviewer’s assessment, disagreements will be resolved through discussion within the review team and, where necessary, by involving additional members. Study selection will be documented using a PRISMA-ScR flow diagram [26].

### Data Charting (Data Extraction)

A structured data charting form will be developed in NVivo (Lumivero) or Microsoft Excel informed by the review objectives and the *JBI Manual for Evidence Synthesis* [25]. This form will be piloted on a small number of included sources prior to full implementation and refined as necessary. The planned data charting items are summarized in Table 3.

**Table 3:** Planned data charting items.

Category	Data items
Bibliographic and general information	Authors, year of publication, and country or region; journal or source type; and funding sources and author-declared conflicts of interest
Study characteristics	Study design (eg, methodological paper, retrospective cohort study, cross-sectional study, qualitative study, mixed methods study, case study, or pilot implementation) and setting and organization type
Population and data sources	Types of safety data used (eg, individual case safety reports, electronic health records, registries, claims data, clinical trial data, published literature, and social media), key patient population or therapeutic area characteristics, and sample size or data volume
Causality assessment level and role	Levels targeted: individual case level, population level, or both and position of the AI-based method within the workflow (eg, information extraction and structuring, application of causality criteria, signal detection and causal interpretation, or governance and reporting)
AI techniques and implementation details	AI methods used (eg, supervised, unsupervised, or reinforcement learning; deep learning; natural language processing; knowledge graphs; rule-based systems; causal models; and hybrid systems), any automation technologies and their role, inputs and features, training and validation data, implementation maturity level (conceptual proposal, prototype or proof of concept, retrospective or prospective validation, pilot implementation, routine deployment, or regulatory or organizational framework), and source type
Information requirements for causality assessment	Types and granularity of the information required at the individual case level (eg, exposure and event timing, de- or rechallenge information, concomitant medications, comorbidities, and laboratory results), information requirements at the population level (eg, exposure populations, comparator groups, follow-up time, confounders, and health care use patterns), and any explicitly stated minimal datasets or mandatory data elements
Data quality and performance metrics	Data quality dimensions (completeness, accuracy, timeliness, consistency, and representativeness) and preprocessing steps; model performance metrics (eg, sensitivity, specificity, PPV^a^, NPV^b^, AUROC^c^, *F*_1_-score, and calibration); validation strategies (internal, cross-validation, temporal, external, and comparison with expert assessment); and fairness, generalizability, or robustness evaluation
Governance and oversight	Human-in-the-loop design (thresholds for manual review, override mechanisms, and escalation pathways); approaches to explainability and transparency; and monitoring, maintenance, and updating (drift detection, retraining schedules, and performance auditing)
Risks and their management	Types of risks discussed (eg, algorithmic bias, model drift, overfitting, misclassification, automation bias, lack of explainability, and accountability and liability) and strategies for risk mitigation
Interaction between individual case– and population-level applications	How outputs from individual-level tools may inform population-level signal detection or causal modeling and vice versa, mechanisms for cross-validation, and shared infrastructure or data pipelines
Main findings and authors’ conclusions	Reported benefits, limitations, and recommendations and identified knowledge gaps and future research directions

^a^PPV: positive predictive value.

^b^NPV: negative predictive value.

^c^AUROC: area under the receiver operating characteristic curve.

Two reviewers will independently chart data from each included source. One reviewer may perform the initial charting, and the second reviewer will verify the entries. Disagreements will be handled using the same 3-reviewer and consensus process used for study selection. The data charting form will remain flexible, and new fields may be added if important themes emerge during the review. Modifications will be reported in the final review.

### Critical Appraisal of Individual Sources of Evidence

In accordance with JBI guidance for scoping reviews, a formal risk-of-bias assessment is not required when the objective is to map existing evidence and concepts [25]. However, given this review’s focus on data quality, validation, and governance, methodological characteristics relevant to interpreting AI-based methods will be documented in a structured manner during data charting. For each included AI-based or prediction model study, the following will be recorded: (1) whether training data provenance is reported, (2) whether the reference standard or labeling process is described, (3) whether subgroup performance is evaluated, (4) whether data leakage controls are described, and (5) whether transportability or generalizability is addressed. These items are informed by the TRIPOD+AI (Transparent Reporting of a Multivariable Prediction Model for Individual Prognosis or Diagnosis for AI studies) reporting guidelines [29] and the PROBAST+AI (Prediction Model Risk-of-Bias Assessment Tool for AI studies) appraisal tool [30], used here as organizing frameworks rather than formal scoring instruments. These methodological characteristics will be reported descriptively in the completed review.

### Data Synthesis and Presentation

Thematic synthesis will follow a reflexive thematic analysis approach [31] using both inductive and deductive strategies. An initial deductive coding framework will be derived from the review’s population, concept, and context framework elements; primary and secondary objectives; and planned data items. Additional themes will be identified inductively. Findings will be presented using a combination of tables, figures (eg, workflow diagrams), and narrative synthesis. Descriptive summaries will be stratified by source type and implementation maturity level to avoid conflating conceptual proposals, prototype systems, validation studies, pilot implementations, and routine deployments. Analysis will be conducted using NVivo, structured Microsoft Excel matrices, or both.

Each analytic product corresponds to a specific research objective: descriptive summaries and thematic findings on requirements and risks address primary objective 1, cross-level comparisons address secondary objective 1, AI technique characterization addresses secondary objective 2, and governance synthesis addresses secondary objective 3.

### Patient and Public Involvement

No patients or members of the public are involved in the design, conduct, reporting, or dissemination of this scoping review as it relies solely on previously published and publicly available data and documents.

## Results

This protocol was registered in the Open Science Framework on December 23, 2025 (osf.io QVF5C) [28]. The registration was subsequently updated on May 19, 2026, to reflect an extension to the data collection period. A preliminary database search was conducted in December 2025, retrieving a total of 760 records, of which the preliminary title and abstract screening identified 196 (25.8%) articles for full-text review. The formal search based on this protocol will be conducted in July 2026. Data charting is scheduled for August 2026, and synthesis is scheduled for September 2026. The completed review is expected to be submitted for publication by the end of December 2026. Deviations from this protocol will be documented and reported in the final review. This research was funded by the Ministry of Health, Labor and Welfare from April 2025 to March 2026 and by the Japan Society for the Promotion of Science from April 2026 to the present.

## Discussion

### Expected Findings

This review is expected to offer several contributions. First, it will provide a structured map of AI-based applications for causality assessment at the individual case and population levels, identifying areas of concentration and evidence gaps. Second, mapping reported information inputs, data quality dimensions, and validation practices is expected to support pharmacovigilance departments and marketing authorization holders when designing or procuring AI-based tools. Third, synthesizing reported risks and mitigation strategies may assist regulators and guideline developers in refining governance frameworks and help AI developers align system designs with pharmacovigilance needs.

Despite these contributions, several limitations are expected. For example, restricting inclusion to English- and Japanese-language sources introduces a potential source of selection bias; relevant work—particularly implementation descriptions and regulatory guidance documents from non–English- or Japanese-speaking regions—may be underrepresented. The broad scope of AI-based methods and the heterogeneous nature of implementations may constrain the ability to compare performance quantitatively. Additionally, the review will be limited by what is documented in the published literature; practical implementation details and internal organizational practices may not be captured.

### Conclusions

This scoping review protocol sets out a systematic and transparent approach to mapping how AI-based methods are used, proposed, and discussed for causality assessment in pharmacovigilance across the individual case and population levels. By consolidating evidence that is currently dispersed across methodological, implementation, and regulatory sources, the planned review is intended to clarify the current landscape and establish a foundation for future empirical and methodological research on AI-supported causality assessment.

## Data Availability

Data sharing is not applicable to this article as no data sets were generated or analyzed during this study.

## Appendix

Multimedia Appendix 1 PRISMA-ScR checklist.

Multimedia Appendix 2 PRISMA-S (Preferred Reporting Items for Systematic Reviews and Meta-Analyses extension for reporting literature searches in systematic reviews) checklist.

Multimedia Appendix 3 Search strategies.

## Abbreviations

JBI: Joanna Briggs Institute
PRISMA-S: Preferred Reporting Items for Systematic Reviews and Meta-Analyses extension for reporting literature searches in systematic reviews
PRISMA-ScR: Preferred Reporting Items for Systematic Reviews and Meta-Analyses extension for Scoping Reviews
PROBAST+AI: Prediction Model Risk-of-Bias Assessment Tool for AI studies
TRIPOD+AI: Transparent Reporting of a Multivariable Prediction Model for Individual Prognosis or Diagnosis for AI studies
