# Supplementary material for: AI for Causality Assessment in Pharmacovigilance: Protocol for a Scoping Review
Source: JMIR Res Protoc. 2026 Jul 16;15:e101691. doi: 10.2196/101691 (PMC13424755; doi:10.2196/101691)
Supplement: Multimedia Appendix 3 [file resprot_v15i1e101691_app3.docx]

**Appendix 3: Search Strategies**

This appendix provides complete search strategies for all five bibliographic databases and supplementary sources. All three blocks are combined using the Boolean operator AND; terms within each block are combined using OR. No publication year filter was applied. Language was restricted to English or Japanese for all database searches.

1. **PubMed (MEDLINE)**

Block 1: Pharmacovigilance

#1 "Pharmacovigilance"[MeSH Terms]

OR "Drug-Related Side Effects and Adverse Reactions"[MeSH Terms]

OR "Adverse Drug Reaction Reporting Systems"[MeSH Terms]

OR "Product Surveillance, Postmarketing"[MeSH Terms]

OR pharmacovigilance[tiab]

OR "drug safety"[tiab]

OR "adverse drug reaction"[tiab] OR "adverse drug reactions"[tiab]

OR "adverse drug event"[tiab] OR "adverse drug events"[tiab]

OR "adverse reaction"[tiab] OR "adverse reactions"[tiab]

OR "drug-related adverse event"[tiab] OR "drug-related adverse events"[tiab]

OR ADR[tiab]

OR "adverse event"[tiab] OR "adverse events"[tiab]

OR "safety signal"[tiab] OR "safety signals"[tiab]

OR "safety report"[tiab] OR "safety reports"[tiab]

OR "spontaneous report"[tiab] OR "spontaneous reports"[tiab]

OR ICSR[tiab] OR ICSRs[tiab]

Block 2: Causality & causal assessment

#2 "Causality"[MeSH Terms]

OR "Bayes Theorem"[MeSH Terms]

OR "Models, Statistical"[MeSH Terms]

OR "causality assessment"[tiab]

OR "causal inference"[tiab]

OR "causal model"[tiab] OR "causal models"[tiab] OR "causal modelling"[tiab] OR "causal modeling"[tiab]

OR "causal relationship"[tiab] OR "causal relationships"[tiab]

OR "signal assessment"[tiab]

OR Naranjo[tiab]

OR "WHO-UMC"[tiab]

Block 3: Artificial intelligence

#3 "Artificial Intelligence"[MeSH Terms]

OR "Machine Learning"[MeSH Terms]

OR "Deep Learning"[MeSH Terms]

OR "Natural Language Processing"[MeSH Terms]

OR "Expert Systems"[MeSH Terms]

OR "artificial intelligence"[tiab]

OR "machine learning"[tiab]

OR "deep learning"[tiab]

OR "natural language processing"[tiab]

OR NLP[tiab]

OR "knowledge graph"[tiab] OR "knowledge graphs"[tiab]

OR "expert system"[tiab] OR "expert systems"[tiab]

OR "prediction model"[tiab] OR "prediction models"[tiab]

OR "generative AI"[tiab]

OR "agentic AI"[tiab]

OR "large language model"[tiab] OR "large language models"[tiab]

OR LLM[tiab] OR LLMs[tiab]

OR ChatGPT[tiab]

OR Claude[tiab]

OR Gemini[tiab]

OR GPT-5[tiab] OR GPT-4[tiab] OR GPT-3[tiab]

Combined strategy

#4 #1 AND #2 AND #3

Language filter

#5 #4 AND (English[lang] OR Japanese[lang])

1. **Web of Science Core Collection**

Block 1: Pharmacovigilance & drug safety

TS=("pharmacovigilance" OR "drug safety" OR "adverse drug reaction*"

OR "adverse drug event*" OR "adverse reaction*"

OR "drug-related adverse event*" OR "ADR"

OR "adverse event*" OR "safety signal*" OR "safety report*"

OR "spontaneous report*" OR "ICSR*")

Block 2: Causality & causal assessment

TS=("causality assessment" OR "causal inference" OR "causal model*"

OR "causal relationship*" OR "signal assessment"

OR "Naranjo" OR "WHO-UMC")

Block 3: Artificial intelligence & automation

TS=("artificial intelligence" OR "machine learning" OR "deep learning"

OR "natural language processing" OR "NLP"

OR "knowledge graph*" OR "expert system*"

OR "prediction model*" OR "generative AI" OR "agentic AI"

OR "large language model*" OR "LLM*"

OR "ChatGPT" OR "Claude" OR "Gemini" OR "GPT-5*" OR "GPT-4*" OR "GPT-3*")

Combined strategy

Block 1 AND Block 2 AND Block 3

Language filter (applied after combination)

Language: English OR Japanese

1. **ProQuest**

Block 1: Pharmacovigilance

ab,ti("pharmacovigilance" OR "drug safety" OR "adverse drug reaction*"

OR "adverse drug event*" OR "adverse reaction*"

OR "drug-related adverse event*" OR "adverse event*"

OR "safety signal*" OR "safety report*" OR "spontaneous report*"

OR "ICSR*" OR "ADR")

Block 2: Causality and causal assessment

ab,ti("causality assessment" OR "causal inference" OR "causal model*"

OR "causal relationship*" OR "signal assessment"

OR "Naranjo" OR "WHO-UMC")

Block 3: Artificial intelligence

ab,ti("artificial intelligence" OR "machine learning" OR "deep learning"

OR "natural language processing" OR "NLP"

OR "knowledge graph*" OR "expert system*"

OR "prediction model*" OR "generative AI" OR "agentic AI"

OR "large language model*" OR "LLM*"

OR "ChatGPT" OR "Claude" OR "Gemini" OR "GPT-5*" OR "GPT-4*" OR "GPT-3*")

Combined strategy

Block 1 AND Block 2 AND Block 3

Language filter

Language: English OR Japanese

1. **EBSCOhost (CINAHL Plus; Academic Search Complete)**

Block 1: Pharmacovigilance

(MH "Pharmacovigilance") OR (MH "Adverse Drug Reaction Reporting Systems")

OR AB("pharmacovigilance" OR "drug safety" OR "adverse drug reaction*"

OR "adverse drug event*" OR "adverse reaction*"

OR "drug-related adverse event*" OR "adverse event*"

OR "safety signal*" OR "spontaneous report*"

OR "ICSR*" OR "ADR")

OR TI("pharmacovigilance" OR "drug safety" OR "adverse drug reaction*"

OR "adverse drug event*" OR "adverse reaction*"

OR "drug-related adverse event*" OR "adverse event*"

OR "safety signal*" OR "safety report*" OR "spontaneous report*"

OR "ICSR*" OR "ADR")

Block 2: Causality and causal assessment

AB("causality assessment" OR "causal inference" OR "causal model*"

OR "causal relationship*" OR "signal assessment"

OR "Naranjo" OR "WHO-UMC")

OR TI("causality assessment" OR "causal inference" OR "causal model*"

OR "causal relationship*" OR "signal assessment"

OR "Naranjo" OR "WHO-UMC")

Block 3: Artificial intelligence

(MH "Artificial Intelligence") OR (MH "Machine Learning")

OR (MH "Natural Language Processing") OR (MH "Expert Systems")

OR AB("artificial intelligence" OR "machine learning" OR "deep learning"

OR "natural language processing" OR "NLP"

OR "knowledge graph*" OR "expert system*"

OR "prediction model*" OR "generative AI" OR "agentic AI"

OR "large language model*" OR "LLM*"

OR "ChatGPT" OR "Claude" OR "Gemini" OR "GPT-4" OR "GPT-3*")

OR TI("artificial intelligence" OR "machine learning" OR "deep learning"

OR "natural language processing" OR "NLP"

OR "knowledge graph*" OR "expert system*"

OR "prediction model*" OR "generative AI" OR "agentic AI"

OR "large language model*" OR "LLM*"

OR "ChatGPT" OR "Claude" OR "Gemini" OR "GPT-5*" OR "GPT-4*" OR "GPT-3*")

Combined strategy

Block 1 AND Block 2 AND Block 3

Language filter

Language: English OR Japanese

1. **Ichushi-Web (医中誌; Japanese medical literature database)**

Block 1: Pharmacovigilance

薬剤疫学 OR ファーマコビジランス OR 医薬品安全性監視

OR 副作用 OR 有害反応 OR 薬物有害反応

OR 有害薬物反応 OR 有害事象

OR 安全性シグナル OR 自発報告

OR ICSR OR 個別症例安全性報告

OR pharmacovigilance OR "adverse drug reaction"

OR "adverse event" OR "drug safety"

Block 2: Causality and causal assessment

因果関係評価 OR 因果評価 OR 因果推論

OR 因果モデル OR シグナル評価

OR ナランホ OR ナランジョ OR Naranjo OR WHO-UMC

OR "causality assessment" OR "causal inference"

Block 3: Artificial intelligence

人工知能 OR 機械学習 OR 深層学習 OR ディープラーニング

OR 自然言語処理 OR ナレッジグラフ OR 知識グラフ

OR エキスパートシステム OR 予測モデル

OR 生成AI OR 大規模言語モデル OR LLM

OR ChatGPT OR "generative AI" OR "agentic AI"

OR "artificial intelligence" OR "machine learning"

OR "natural language processing"

Combined strategy

Block 1 AND Block 2 AND Block 3

Language filter: No restriction (Ichushi-Web is a Japanese-language medical literature database; no language restriction is applied.)

1. **Supplementary Searches**

6.1 Reference list screening

Reference lists of all included studies and relevant systematic or scoping reviews, guidelines, and consensus documents identified during the search will be screened to identify further eligible sources.

6.2 Forward citation tracking

Where appropriate, forward citation tracking will be conducted for key included studies using Web of Science or Google Scholar.

6.3 Targeted searches of regulatory and organizational websites

The following regulatory and organizational websites will be searched to identify technical reports, guidance documents, or position papers describing AI-based causality assessment systems that may not be indexed in bibliographic databases:

- European Medicines Agency (EMA): <https://www.ema.europa.eu>
- U.S. Food and Drug Administration (FDA): <https://www.fda.gov>
- World Health Organization (WHO): <https://www.who.int>
- CIOMS (Council for International Organizations of Medical Sciences): <https://cioms.ch>
- Uppsala Monitoring Centre (UMC): <https://who-umc.org>
- Pharmaceuticals and Medical Devices Agency (PMDA, Japan): <https://www.pmda.go.jp>

Searches will be conducted using the site-specific search function.

Key terms used: "artificial intelligence", "causality assessment", "pharmacovigilance", "adverse drug reaction", and their equivalents.

1. **PRISMA-S Reporting**

This search strategy has been developed and reported in accordance with the Preferred Reporting Items for Systematic Reviews and Meta-Analyses extension for reporting literature searches (PRISMA-S) [Rethlefsen ML, et al. Syst Rev. 2021;10:39]. The completed PRISMA-S checklist is provided as Appendix 3.

**Abbreviations**

AB: abstract field

ADR: adverse drug reaction

ICSR: individual case safety report

LLM: large language model

MeSH: Medical Subject Headings

MH: subject heading field (EBSCOhost)

NLP: natural language processing

TI: title field

tiab: title/abstract field (PubMed)

TS: topic field (Web of Science)

WHO-UMC: World Health Organization–Uppsala Monitoring Centre
